# Melatonin and hyperbaric oxygen therapies suppress colorectal carcinogenesis through pleiotropic effects and multifaceted mechanisms

**DOI:** 10.7150/ijbs.62280

**Published:** 2021-08-27

**Authors:** Yi-Chen Li, Chih-Hung Chen, Chia-Lo Chang, John Yi-Wu Chiang, Chi-Hsiang Chu, Hong-Hwa Chen, Hon-Kan Yip

**Affiliations:** 1 Division of Cardiology, Department of Internal Medicine, Kaohsiung Chang Gung Memorial Hospital and Chang Gung University College of Medicine, Kaohsiung 83301, Taiwan;; 2 Clinical Medicine Research Center, National Cheng Kung University Hospital, College of Medicine, National Cheng Kung University, Tainan 70403, Taiwan;; 3 Center of Cell Therapy, National Cheng Kung University Hospital, College of Medicine, National Cheng Kung University, Tainan 70403, Taiwan;; 4 Divisions of General Medicine, Department of Internal Medicine, Kaohsiung Chang Gung Memorial Hospital and Chang Gung University College of Medicine, Kaohsiung 83301, Taiwan;; 5 Division of Colorectal Surgery, Department of Surgery, Kaohsiung Chang Gung Memorial Hospital and Chang Gung University College of Medicine, Kaohsiung 83301, Taiwan; 6 Department of Computer Science & Engineering, National Sun Yat-Sen University, Kaohsiung 80424, Taiwan;; 7 Department of Healthcare Administration and Medical Informatics, Kaohsiung Medical University, Kaohsiung 80708, Taiwan;; 8 Department of Statistics, Tunghai University, Taichung 40704, Taiwan;; 9 Center for Shockwave Medicine and Tissue Engineering, Kaohsiung Chang Gung Memorial Hospital, Kaohsiung 83301, Taiwan;; 10 Institute for Translational Research in Biomedicine, Kaohsiung Chang Gung Memorial Hospital, Kaohsiung 83301, Taiwan;; 11 Department of Nursing, Asia University, Taichung 41354, Taiwan;; 12 Department of Medical Research, China Medical University Hospital, China Medical University, Taichung 40402, Taiwan;; 13 Division of Cardiology, Department of Internal Medicine, Xiamen Chang Gung Hospital, Xiamen 361028, Fujian, China.

**Keywords:** colorectal cancer, hyperbaric oxygen, melatonin, cancer growth, invasion, migration, cancer stem cell, carcinogenesis

## Abstract

Colorectal cancer (CRC) is the third most common cancer worldwide. Colorectal carcinogenesis is frequently induced by hypoxia to trigger the reprogramming of cellular metabolism and gain of malignant phenotypes. Previously, hyperbaric oxygen (HBO) therapy and melatonin have been reported to alter the hypoxic microenvironment, resulting in inhibiting cancer cell survival. Accordingly, this study tested the hypothesis whether HBO and melatonin effectively inhibited CRC carcinogenesis. In vitro results indicated that melatonin therapy significantly suppressed the malignant phenotypes, including colony formation, growth, invasion, migration and cancer stemness with dose-dependent manners in CRC cell lines through multifaceted mechanisms. Similar to in vitro study, in vivo findings further demonstrated the melatonin, HBO and combined treatments effectively promoted apoptosis (cleaved-caspase 3/ cleaved-PARP) and arrested tumor proliferation, followed by inhibiting colorectal tumorigenesis in CRC xenograft tumor model. Moreover, melatonin, HBO and combined treatments modulated multifaceted mechanisms, including decreasing HIF-1α expression, alleviating AKT activation, repressing glycolytic metabolism (HK-2/PFK1/PKM2/LDH), restraining cancer stemness pathway (TGF-β/p-Smad3/Oct4/Nanog), reducing inflammation (p-NFκB/ COX-2), diminishing immune escape (PD-L1), and reversing expression of epithelial mesenchymal transition (E-cadherin/N-cadherin/MMP9). In conclusion, melatonin and HBO therapies suppressed colorectal carcinogenesis through the pleiotropic effects and multifaceted mechanisms, suggesting melatonin and HBO treatments could be novel therapeutic strategies for CRC treatment.

## Introduction

According to World Health Organization GLOBOCAN database, the colorectal cancer (CRC) is the third most common cancer in males and second in females, with 1.8 million new cases and about 881,000 deaths globally in 2018. Regrettably, the 5-year survival rate for CRC with late stages is low 1. Plentiful data have shown that during tumor progression, CRC cancer cells profoundly interact with their surrounding environment (i.e., tumor microenvironment) that ultimately determines whether the tumor is eliminated, metastasizes or event recurrent 2. The basic research has further revealed that hypoxia is one of the extreme factors that influences the CRC microenvironment 2. Additionally, the rapid proliferation of CRC, resulting in rapid exhaustion of oxygen supply in the absence of vascular supply, is a common causal contributor to create the hypoxic microenvironment in CRC 3. Intriguingly, to adapt for hypoxic stress, cancer cells predominantly produce energy by glycolysis rather than by oxidative decarboxylation, followed by lactic acid fermentation, which is called the Warburg effect 4, 5. Hypoxia inducible factor-1 (HIF-1) is a transcriptional regulator responded to hypoxia to modulate glycolytic enzymes 6 7, that is considered as a poor prognostic factor of cancer and is a malignant contributor of metabolic alteration, angiogenesis, invasiveness, therapeutic resistance, immune escape, and resistance to cell death 8. Thus, hypoxia plays a central role in tumor progression and recurrence. Hence, it is well suggested as the potential target in cancers 9.

Melatonin (N-acetyl-5-methoxytryptamine), a small molecular hormone, is mainly produced by the pineal gland in all vertebrates and releases according to circadian rhythms 10. Additionally, melatonin is recognized as a free radical scavenge as well as possesses strong capacity of anti-inflammation and antioxidant effects through increasing the efficiency of the electron transport chain and reducing electron leakage to decease the generation of free radicals 11-15. Previous literatures indicate that melatonin has strongly oncostatic effects in colorectal cancer, hepatic cancer, pancreatic cancer, lung cancer, breast cancer, ovarian cancer, prostate cancer, glioblastoma, bone cancer, and leiomyosarcoma 16. Another reports demonstrated that melatonin suppresses HIF-1 transcriptional activity which, in turn, ameliorates tumor angiogenesis under hypoxia condition in the CRC cell line 17. Moreover, melatonin stimulates apoptosis, inhibits pro-survival signal, suppresses angiogenesis, and modulates tumor metabolism in cancer cells 18-22, suggesting melatonin has therapeutic potential on cancers, including those of CRCs.

On the other hand, hyperbaric oxygen (HBO) therapy is an alternative treatment for some disease entities such as peripheral arterial occlusive disease that patients breathe 100% oxygen while being exposed to increased atmospheric pressure 23. The HBO treatment would increase the amount of dissolved oxygen in blood and thereby escalate O_2_ delivery to tissues for the purpose of improving those of disease-related hypoxia and ischemia 24. Of special interest is that the previous studies, i.e., in vitro or in vivo data, revealed that the HBO treatment suppressed the tumor growth in leukemia, mammary, ovarian and glioma cancers 25-28. However, the HBO treatment has been reported insufficiently suppressed the tumor growth in lung cancer, prostate cancer, as well as head and neck cancer 29-31. So far, HBO treatment to cancer has still lacked a solid conclusion, suggesting the need of further study to determine the role of HBO treatment in cancer therapy, especially when its accessory therapeutic role is taken into consideration, i.e., combined with other therapy, called the cocktail therapy, to upgrade the therapeutic effect of HBO.

Undoubtedly, tumorigenesis is frequently induced by the accumulations of oncogenic mutations and hypoxia, which further trigger the alterations of cellular metabolism and microenvironment 32. On the other hand, both HBO and melatonin have been shown as potential candidates of anti-tumor ability and reprogramming of cellular metabolism 24, 33; however, there is no evidence about combined melatonin and HBO therapy in cancer treatment. Based on aforementioned premise, we proposed that combined melatonin and HBO (i.e., cocktail therapy) might be superior to either one alone for reducing the colorectal carcinogenesis.

## Materials and methods

### Cell culture

The colorectal cancer cell lines, DLD-1 and LoVo, were purchased from Bioresource Collection and Research Center, Taiwan. DLD-1 cells (Dukes' type C, human colorectal adenocarcinoma) were incubated in high glucose Dulbecco's modified Eagle's medium (Gibco) supplemented with 10% fetal bovine serum (Gibco) and 1% penicillin/streptomycin (Gibco). The LoVo cells (Dukes' type C, grade IV, human colorectal adenocarcinoma) were grown in DMEM/F12 medium (Gibco) contained with 10% fetal bovine serum and 1% penicillin/streptomycin. All cultured cells were maintained at 37C and 5% CO2.

### Assessment of cell viability, colony formation and sphenoid formation

As previously described 34, the MTS assay was performed to determine cell viability. About 3000 cells in 100 ul of medium were seeded into 96-well plates and incubated for the indicated time. At the end of incubation, MTS reagent (Promega) was added to samples, incubated at 37 °C for 2hr and detected by 490 μm length absorption by ELISA reader. Additionally, for colony formation assay, cells were plated into a 6-well plate, cultured for 7~14 days and then stained by Giemsa Modified Solution (Sigma). Cell spheroid formation was analyzed by culturing cells for 3 weeks in a suspension with serum free medium containing N2 supplement (Invitrogen), 10 ng/ml EGF (Invitrogen), and 10 ng/ml bFGF (Invitrogen). After 3 weeks, cellular spheres were visualized and counted with 10 independent microscopic fields each sample.

### Flow cytometric analysis for apoptosis

The percentages of viable and apoptotic cells were stained by double staining of annexin V and propidium iodide (BD Biosciences) and calculated by flow cytometry (Beckman Coulter) based on the previous study 35. A minimum of 10,000 cells were then analyzed by Cell Quest software (Beckton Dickinson).

### Wound-Healing Migration Assay

For the wound-healing migration assay, cells were plated onto culture inserts (iBidi GmbH). After full confluence of culture cells, the inserts were removed and treated with or without melatonin and HBO treatments for migration assay, respectively. After 24 hr incubation, the migration of cells into the denuded areas in the marked region was monitored. To measure migration, cells were captured with microphotography, and the total migration distance was analyzed using Image J software (National Institutes of Health).

### In vitro invasion assay

According to the previous study 36 , the invasion assay was performed using a chemotaxis chamber (8-um pores, 6.5-mm diameter, Corning) with a matrix-coated polycarbonate filter. Cells were seeded in the upper well of each chamber compartment. Twenty percentage of FBS medium serving as the chemoattractant was loaded into the lower chamber at 37°C with 5% CO2. After 24 hr, migrated cells in the underside were fixed by methanol and further stained by Giemsa solution, while non-migrated cells were removed from the upper chamber.

### Reverse transcription quantitative polymerase chain reaction (RT-qPCR)

Total RNA was extracted by miRNeasy Mini kit (QIAGEN), and reverse transcription from RNA to cDNA was performed by high-Capacity cDNA Reverse Transcription Kit (Applied Biosystems). PCR was analyzed on Step One‐Plus (Applied Biosystems) PCR detection system using QuantiNovaTM SYBR^R^ Green PCR Kit (Applied Biosystems). The primers used in this study were listed in the Supplementary Table 1.

### Cellular protein extraction and western blot analysis

Western blot protocols were based on our previous study 37. The 50 ug protein extracts were loaded to SDS-PAGE gel and then transferred to a polyvinylidene difluoride membrane (GE). After blocking overnight, the membranes were incubated by optimal primary antibodies and then horseradish peroxidase-conjugated secondary antibodies to detect the signal by chemiluminescence (Millipore) and Biomax L film (Kodak). All antibody information was listed in supplementary Table 2. The semi-quantified signals were evaluated by Labwork software (UVP, Waltham).

### Xenograft tumor model of nude mice in vivo

The pathogen-free and 4-5 weeks old male BALB/c nude mice were purchased from Charles River Technology (BioLASCO, Taiwan). For tumorigenesis study, A total of 2×10^6^ DLD-1 cells with luciferase activity were subcutaneously injected in 100 ul PBS to the right back of nude mice and detected by IVIS In Vivo Imaging System (Caliper, PerkinElmer). After 7 days or around 100 mm^3^ of tumor size, the mice were divided into four groups: Group 1 (injection of tumor cells without treatment, control group), Group 2 (injection of tumor cells with melatonin 50 mg/kg/day I.P. treatment every day until euthanasia end point), Group 3 ( injection of tumor cells with 1.5 hr/time/day HBO for three times in a weak until euthanasia end point), and Group 4 (injection of tumor cells with combined treatment of HBO and melatonin therapies). The dosage of the melatonin utilized in the present study was based on our previous report with some modification 38. Additionally, the HBO therapy in the current study was based on our another report 39.

The tumor size and body weight of mice were measured twice a week. All animal experimental procedures were approved by the Institute of Animal Care and Use Committee at Kaohsiung Chang Gung Memorial Hospital (Affidavit of submission of Animal Use Protocol No. 2017052301) and performed according to the Eighth Edition of the Guide for the Care and Use of Laboratory Animals (NRC 2011). Mice were housed in an Association for Assessment and Accreditation of Laboratory Animal Care International (AAALAC)-approved animal facility of our hospital with controlled temperature at 24°C and 12 hr light/dark cycle with lights on at 7 a.m.

### Hyperbaric oxygen (HBO) therapy

The procedure of HBO therapy was based on our previous report 40. Briefly, to elevate tissue-level hyperoxia, mice were subjected to HBO administration in an animal tabletop chamber (Piersol-Dive) with the exposure to 100% oxygen at 2.4 atmospheres absolute (ATA) for 90 minutes (1.5 hr/session).

### Immunohistochemical staining

Immunohistochemical staining protocols were based on our previous study 41. Briefly, paraffin sections were treated with 3% H_2_O_2_ for 10 minutes and incubated with Immuno-Block reagent (BioSB) for 30 minutes at room temperature, followed by incubation with the primary antibody specifically against Ki67 (Abcam) and secondary antibody. Immunoreactive signal was visualized by enhanced DAB kit (Abcam).

### Statistical analysis

GraphPad Prism software (GraphPad) was used for statistical analyses. Quantitative data were expressed as mean ± SD. Comparisons between groups were analyzed by one-way or two-way ANOVA, followed by Turkey's comparison. A probability value <0.05 was considered statistically significant.

## Results

### The effect of melatonin significantly suppressed on cellular growth and colony formation and induced on apoptosis in CRC cells through inhibiting AKT signaling pathway

**Figure 1:**
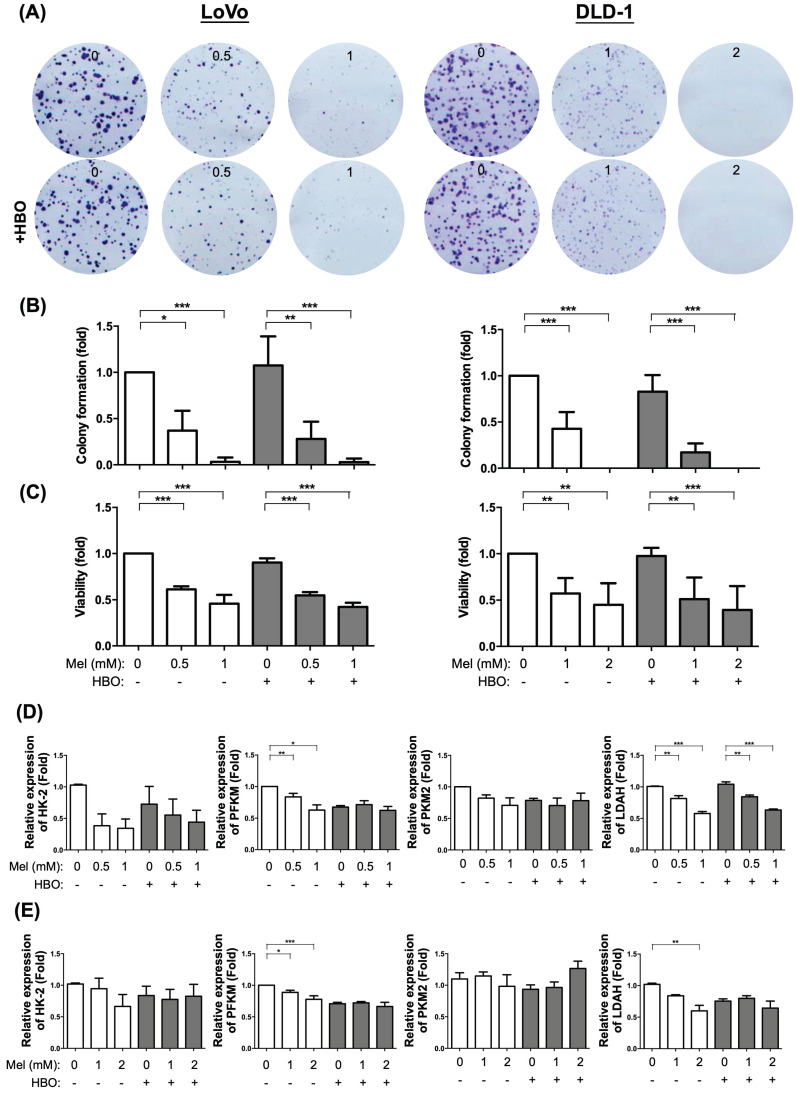
** The effect of melatonin was significantly suppressed on cancer growth in both LoVo and DLD-1 cell lines of the colorectal cancer.** (A) The CRC cells, LoVo and DLD-1, were treated with different concentrations of melatonin (0~2 mM) for 48 hr or underwent HBO exposure once with 100% oxygen at 2.4 ATA for 90 minutes. The original colony pictures were shown. (B) The quantification result of colony formation was presented. (C) After treating with melatonin or HBO, the cellular growth or viability was performed by MTS assay. (D) The mRNA expressions of HK-2, PFKM, PKM2, and LDAH were measured by RT-qPCR in LoVo cell line. (E) The mRNA expressions in DLD-1 cell line. Data represents the analysis of 3~4 times of independent experiments and shows mean ± SD. Mel: melatonin treatment; HBO: hyperbaric oxygen; * represents p<0.05; ** indicates p<0.001; *** shows p<0.0001.

Based on the premise that the typical hallmark of cancer is unlimited proliferation through evading growth suppressors and resisting cell death 42, we first evaluated and examined the melatonin and HBO therapies in inhibiting cancer growth and colony formation ability. The colorectal cancer cells were treated by different concentrations of melatonin (0~2 mM) with or without HBO therapy in both LoVo and DLD-1 colorectal cancer cell lines. The result demonstrated that colony formation and cancer growth were significantly suppressed by melatonin with dose-dependent manner in both LoVo and DLD-1 cell lines (Figure 1A-C). Surprisingly, the HBO therapy did not match the effect of melatonin on suppressing the LoVo and DLD-1 cell lines (Figure 1A-C).

It is well recognized that Warburg effect supports a metabolic environment, which allows for the rapid biosynthesis to sustain tumor growth and proliferation 43. In the present study, we found that the mRNA expressions of hexokinase 2 (HK2), Phosphofructokinase (PFKM), pyruvate kinase muscle isozyme 2 (PKM2), lactate dehydrogenase A (LDHA), four indices of aerobic glycolysis, were down-regulated by melatonin. Especially, the mRNA expressions of PFKM and LDHA were significantly reduced in both DLD-1 and LoVo cell lines, suggesting that melatonin attenuated tumor growth via regulating the re-programming of tumor metabolism.

**Figure 2:**
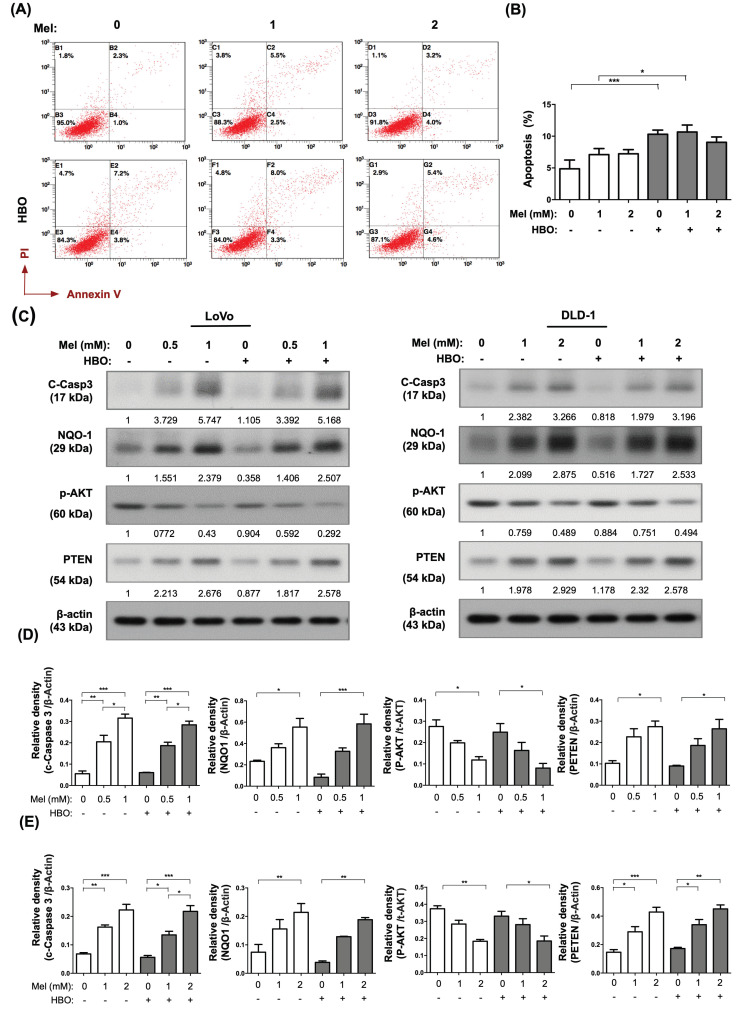
** The effect of melatonin was remarkably induced on apoptosis occurrence in CRC cells.** (A) The apoptosis was measured by Annexin-V/PI staining. The original analysis of flowcytometry was shown. (B) The quantification of apoptotic result was presented. (C) Western blotting was used to detect the protein levels of cleaved Caspase-3, NQO-1, phosphorylated-AKT, and PTEN in LoVo and DLD-1 cell lines. The average densitometry readings/intensity ratio was normalized by control and shown below. (D) The quantification of original densitometry readings/intensity ratio in LoVo cell line. (E) The quantification of original densitometry readings/intensity ratio in DLD-1 cells. Data represents the analysis of 3~4 times of independent experiments and shows mean ± SD. Mel: melatonin treatment; HBO: hyperbaric oxygen; * represents p<0.05; ** indicates p<0.001; *** shows p<0.0001.

We further found that molecular-cellular levels of apoptosis were augmented by melatonin treatment which were measured by Annexin-V/PI staining (i.e., cellular level) and measured by Western blot for cleaved-caspase 3 (i.e., molecular level) (Figure 2A-C). AKT pathway is a critical survival pathway in colorectal cancer 44. Meanwhile, the protein expression of PTEN was up-regulated, whereas the AKT activation was down-regulated by melatonin treatment in both cell lines (Figure 2C-E). On the other hand, the NQO-1 protein level was elevated by melatonin treatment, suggesting that melatonin had an antioxidant property (Figure 2C-D). Interestingly, cellular apoptosis was significantly enhanced by HBO therapy in DLD-1 cell line (Figure 2A). Taken together, the effects of melatonin were not only remarkably to suppress tumor growth but also illustriously triggered tumor apoptosis through inhibiting AKT activation as well as promoting PTEN signaling pathway.

### The effect of melatonin remarkably suppressed on the migration and invasion of CRC cells via TGF-β/Smad3 signaling axil

**Figure 3:**
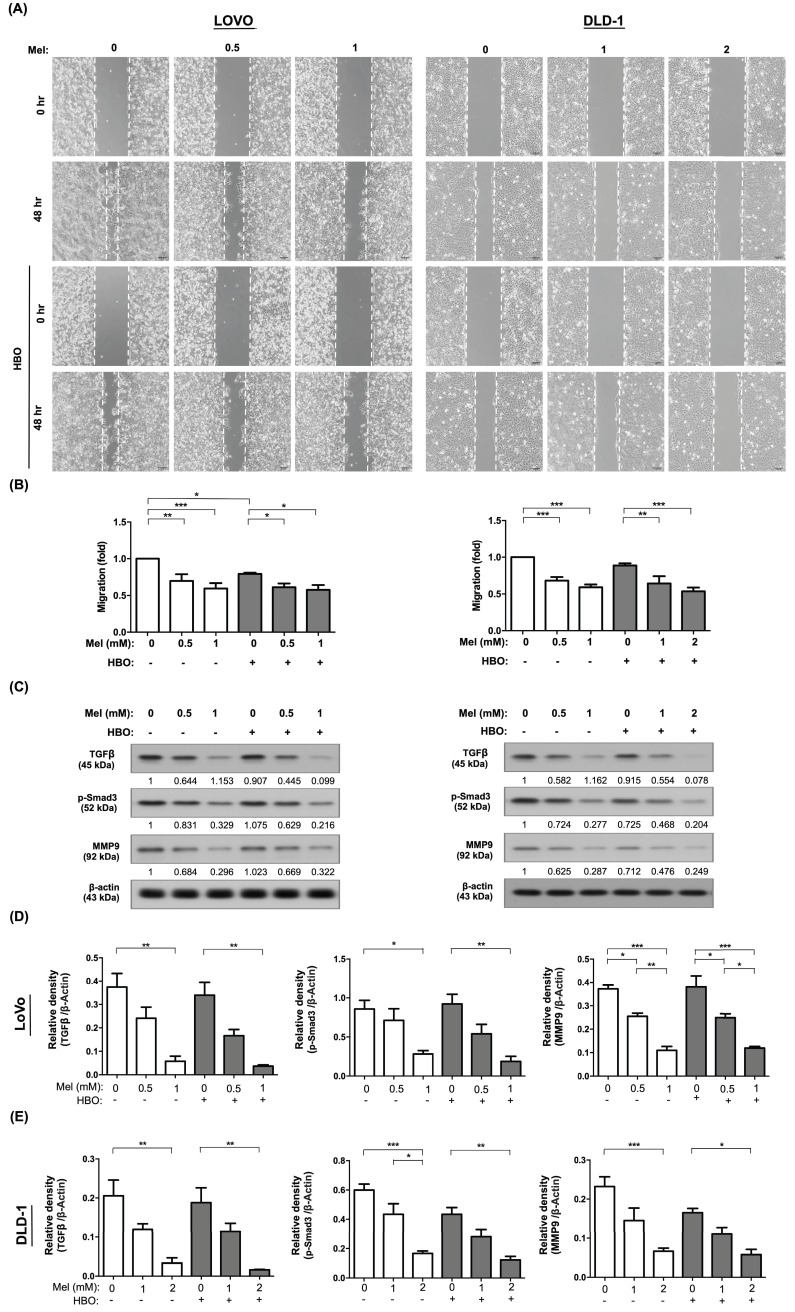
** The effect of melatonin was notably blocked on migratory abilities via TGF-β/Smad3 signaling axil in CRC cells.** (A) Cellular migratory ability was evaluated by wound-healing migration assay. The original pictures were shown. (B) The quantification result of migration was presented. (C) Western blotting was performed to evaluate the protein levels of TGF-β, phosphorylated-Smad3, and MMP9 in LoVo and DLD-1 cell lines. (D) The quantification of original densitometry readings/intensity ratio in LoVo cell line. (E) The quantification of original densitometry readings/intensity ratio in DLD-1 cells. Data represents the analysis of 3~4 times of independent experiments and shows mean ± SD. Mel: melatonin treatment; HBO: hyperbaric oxygen; * represents p<0.05; ** indicates p<0.001; *** shows p<0.0001.

**Figure 4:**
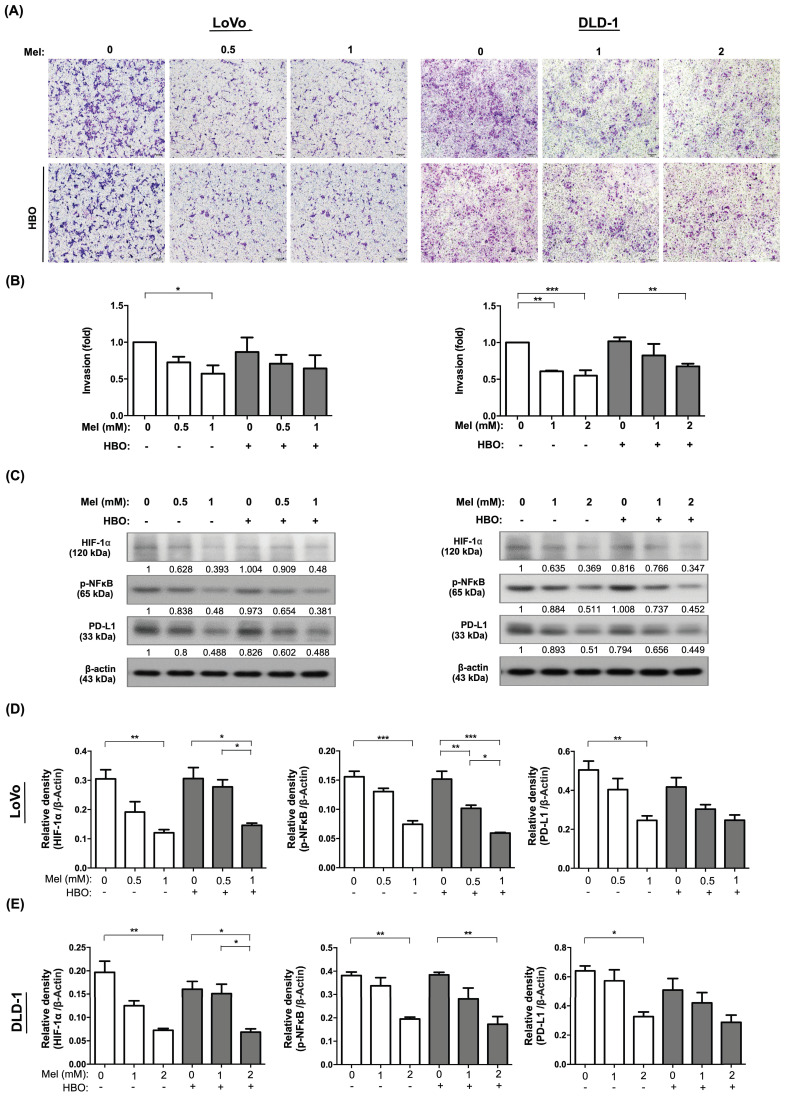
** The effect of melatonin was prominently reduced on invasive properties in CRC cells.** (A) Cellular invasive ability was evaluated by transwell Matrigel invasion assay. The original pictures were shown. (B) The quantification result of invasion was presented. (C) Western blotting was used to detect the protein levels of HIF-1α, phosphorylated-NFκB, and PD-L1 in LoVo and DLD-1 cell lines. (D) The quantification of Western blotting result in LoVo cell line. The average densitometry readings/intensity ratio was normalized by control and shown below. (E)The quantification of original densitometry readings/intensity ratio in DLD-1 cells. Data represents the analysis of 3~4 times of independent experiments and shows mean ± SD. Mel: melatonin treatment; HBO: hyperbaric oxygen; * represents p<0.05; ** indicates p<0.001; *** shows p<0.0001.

Tumor cells acquire migratory phenotypes and digest the extracellular matrix to invade from the primary tumor and extend to native tissues/organs, and finally to form metastasis through TGF-β/Smad3 signaling axil 45. We next explored the effects of melatonin and HBO on inhibiting the cancer cell migration. Through evaluating migration by wound-healing assay, the effect of melatonin on inhibiting the cellular migration was clearly identified with a dose-dependent manner in both LoVo and DLD-1 cancer cell lines (Figure 3 A-B). Surprisingly, the effect of HBO therapy was recognized to augment melatonin on further suppression of this migratory inhibition in LoVo cell line, but not in DLD-1 cell lines (Figure 3 A-B). Moreover, we analyzed the invasion abilities of these cancer cells by Matrigel invasion assay. The results showed that the effect of melatonin on invasion ability was extremely reduced in absence of melatonin treatment (Figure 4A-B). On the other hand, the effect of HBO therapy on invasive ability didn't show any difference between with and without HBO therapy.

We further explore the fundamental mechanism of melatonin and HBO therapies on suppressing the colorectal cancer. The molecular level was evaluated by using the Western blot. As we expected, the protein expressions of TGF-β and Smad3 were remarkably downregulated by melatonin treatment in both LoVo and DLD-1 cells, which further diminished the downstream protein expression of MMP9 (Figure 3 C-E). This result revealed that the effects of melatonin on migration and invasion were mainly via inhibition of TGF-β/Smad3/MMPs signaling axil.

On the other hand, tumor hypoxia and inflammatory situation frequently mediate cancer progression and cancer immune escape 46, 47. Importantly, we found that the protein levels of transcription factor families, including hypoxia inducible factor-1α (HIF-1α) and phosphoryl nuclear factor of κ-light-chain-enhancer of activated B cells (p-NFκB) were significantly attenuated by melatonin treatment, followed by downregulation of programmed death-ligand 1 (PD-L1). Hence, our result demonstrated that the effect of melatonin was remarkably through modulation of multifaceted mechanisms to restrain cancer malignant phenotypes, including tumor growth, migration and invasion.

### The effect of melatonin was notably on ameliorating cancer stem cell properties and associated molecular expression

**Figure 5:**
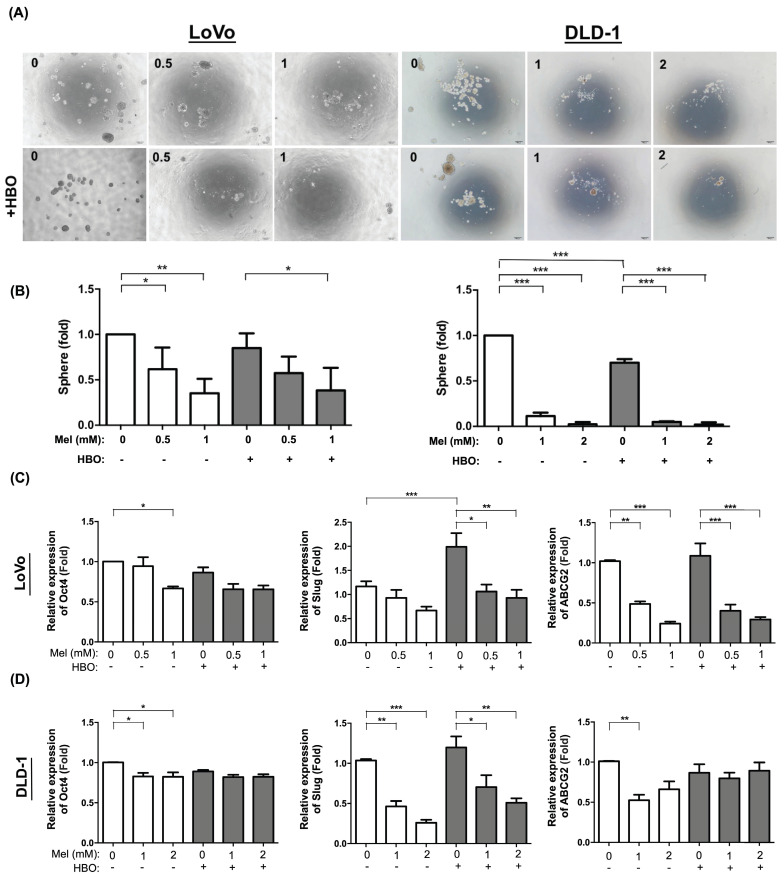
** The effect of melatonin was markedly inhibited on sphere forming abilities in CRC cells.** (A) The sphere forming ability was evaluated from single cell to form single sphere formation. The original pictures were shown. (B) The quantification result of sphere formation was presented. (C) The mRNA levels of cancer stemness-associated molecules, including Oct-4, Slug and ABCG2, were detected by RT-qPCR analysis in LoVo cell line. (D) The mRNA expressions in DLD-1 cell line. Data represents the analysis of 3~4 times of independent experiments and shows mean ± SD. Mel: melatonin treatment; HBO: hyperbaric oxygen; * represents p<0.05; ** indicates p<0.001; *** shows p<0.0001.

Recent study hypothesizes that cancer stem cells (CSCs) provide a small fraction of progenitor cells to undergo neoplastic progression and metastasis 48. To determine self-renewal ability which is a cardinal feature of CSCs, we evaluated sphere forming ability from single cell to form the sphere in non-adherent cultures 49. The result demonstrated that as compared without melatonin and HBO, sphere forming ability was significantly inhibited by melatonin with the dose-dependent manner in both LoVo and DLD-1 cell lines. The effect of HBO therapy was detected to furthermore enhance the inhibition of melatonin in DLD-1 cell line but not in LoVo cell line (Figure 5 A-B).

In the present study, the cancer stem cells were found to possess the self-renewal ability in a de-differentiated state to generate heterogeneous bulk tumors. The transcription factors, including OCT4 (POU5F1) and Slug, regulate the pluripotency circuitry and involve in CSCs conversion. OCT4 further induces the activation of ABCG2, a membrane transport channel, which contributes to chemoresistance 50. In this way, we also found the mRNA levels of cancer stemness-associated molecules, including POU5F1, Slug and ABCG2, were significantly decreased by melatonin treatment in both LoVo and DLD-1 cell lines. The effect of HBO therapy did not enhance on the suppression of melatonin in these cell lines (Figure 5 D-E). In summary, the effect of melatonin suppression of cancer malignant properties might be through the inhibition of cancer stemness and associated molecular levels.

### The effects of melatonin and HBO therapies on suppressing the cancer stemness regulators was fundamentally through suppression of the tumorigenesis in vivo

To address the efficacy of melatonin and HBO therapies in vivo, we established a xenografted luciferase-tagged tumor model for in vivo imaging system (IVIS) in BALB/C nude mice. The colorectal cancer cells, DLD-1, were injected subcutaneously into the back of mice. After 7 days, melatonin treatment was intraperitoneally administered into group 2 and group 4 of mice with 50 mg/kg/day dose until euthanasia end point. Meanwhile, 2 atm HBO treatment for 1.5 hours per section, three times a week, was performed in groups 3 and 4 animals.

**Figure 6:**
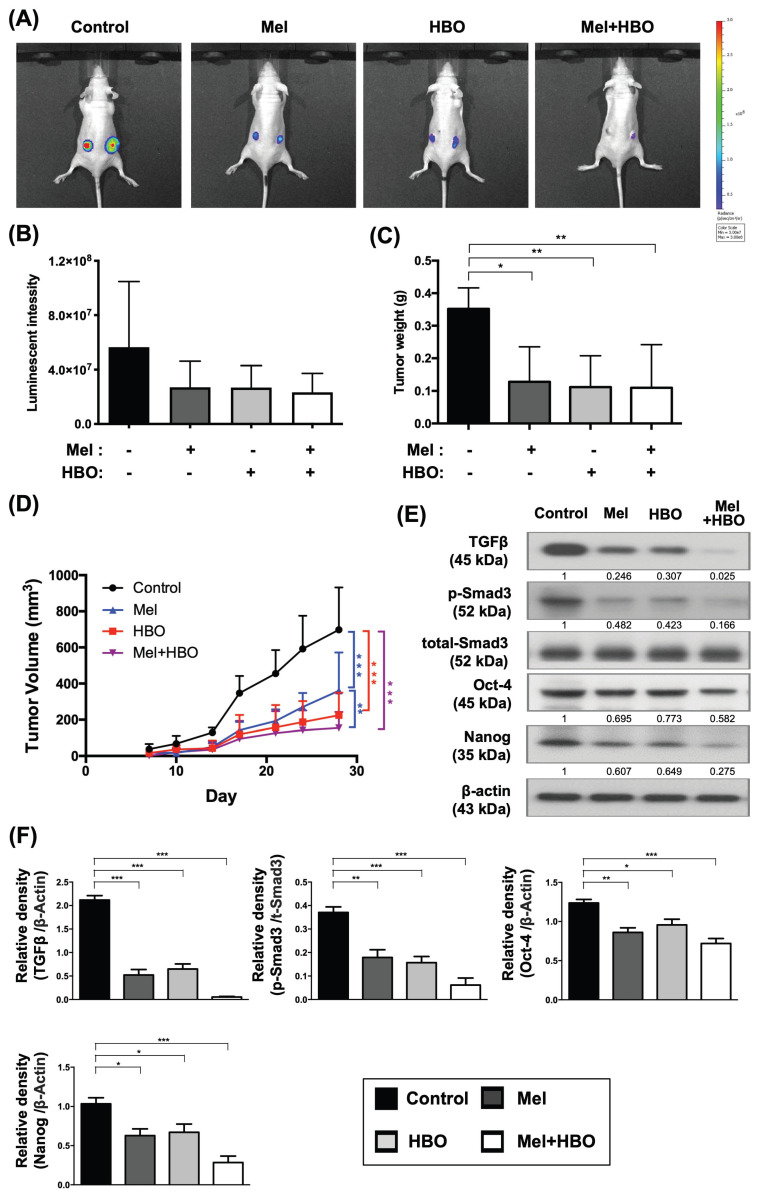
** The effects of melatonin and HBO therapy were significantly reduced on cancer stemness regulators to effectively suppress tumorigenesis in vivo.** The DLD-1 cells were subcutaneously injected into the flanks of nude mice to generate tumors. (A) The size of tumor was monitored by IVIS Imaging System in xenograft tumor model. (B) The bioluminescent signals of tumor cells were measured by IVIS system and significant differences among groups (p=0.047). (C) At the end point of euthanasia, tumor weights were calculated and significant differences among groups (p=0.041). (D) The tumor volumes were measured twice a week for 28 days to evaluate tumorigenesis. (E) Western blotting was performed to evaluate the protein levels of TGF-β, phosphorylated-Smad3, total-Smad3, Oct-4, and Nanog in CRC tumors. The average densitometry readings/intensity ratio was normalized by control and shown below. (F) The quantification of original densitometry readings/intensity ratio. n = 5 for each group. Data represents the analysis and shows mean ± SD. Mel: melatonin; HBO: hyperbaric oxygen; * represents p<0.05; ** indicates p<0.001; *** shows p<0.0001.

By day 28 after tumor injection, luminescent signals of tumor shown in Fig. 6A-B were noticeably decreased in melatonin (group 2), HBO (group 3), and combined treatments (group 4), when compared with tumor only control (group 1) . Tumor weight significantly reduced to about 35% in group 1-3 (Fig. 6C). Additionally, tumor volumes were significantly declined, 48%, 68 %, and 78 % in group 2, group 3 and group 4, respectively, when compared to the group1, suggesting that the effects of melatonin, HBO and combined treatments significantly restrained on tumorigenesis (Figure 6D).

Based on the result of the in vitro study, we further explored the cancer stemness associated regulators. As our expected, the protein expressions of cancer stemness associated regulators, including TGF-β, Oct-4, Nanog, and p-Smad3 activation, were significantly reduced 30~97% by melatonin, HBO, and combined treatments (Figure 6E-F). Since cancer stemness associated regulators played the critical role in tumor initiation and tumorigenesis, these results indicated that the effects of melatonin and HBO therapies substantially prohibited on cancer stemness regulators, as a consequence, to effectively suppress tumorigenesis.

### The effects of melatonin and HBO therapies were clarified to prominently suppress on tumor proliferation and promoted apoptosis through multifaceted mechanisms

**Figure 7:**
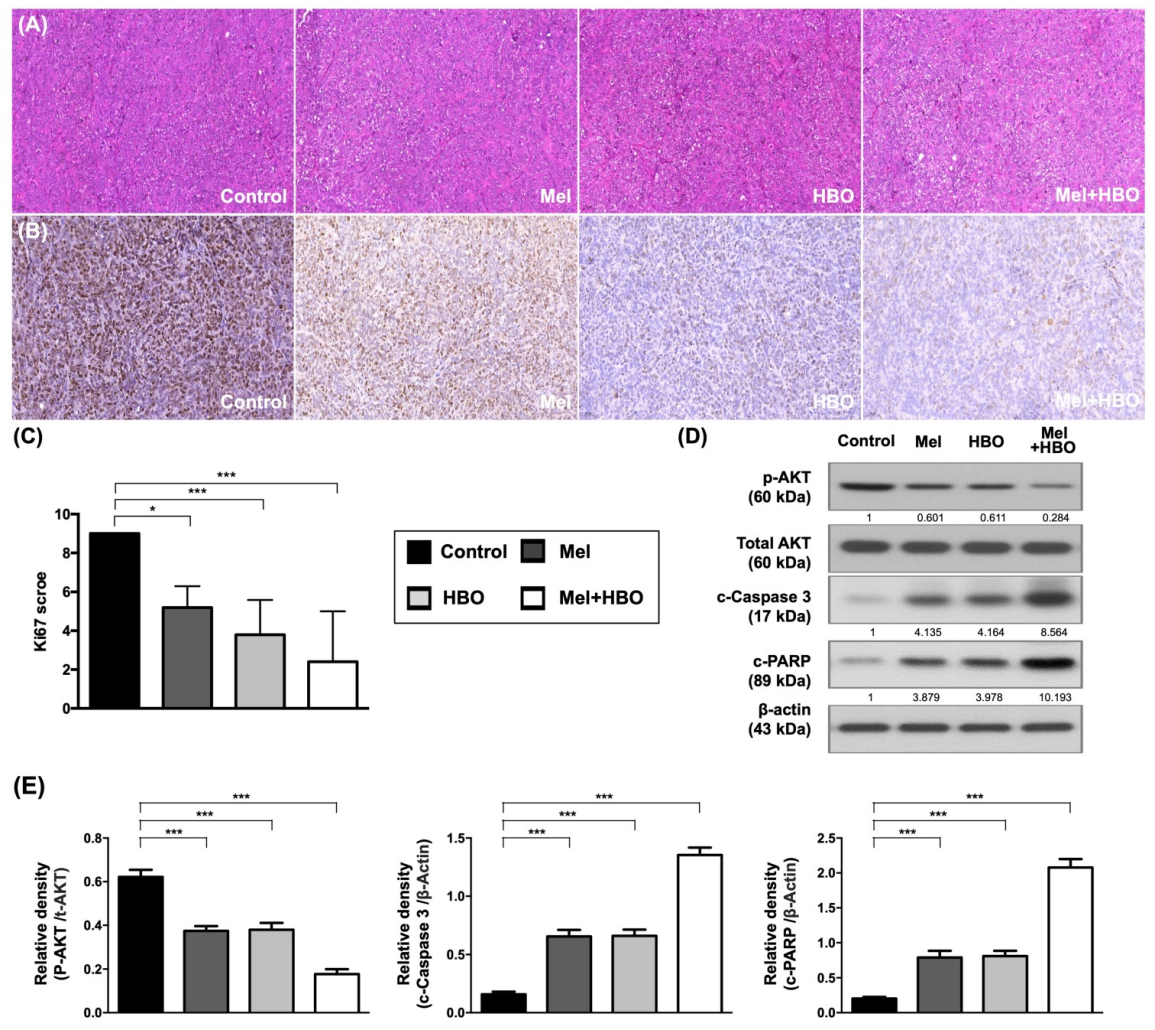
** The effects of melatonin and HBO therapy were prominently suppressed on tumor proliferation and promoted on apoptosis through attenuating AKT activation.** (A) Histology of the tumor tissue was observed by hematoxylin and eosin staining in each group (200X). Scale bars: 50 μm (B) Representative images (200x) of each group for validation of positively stained Ki67 (brown color) by immunohistology staining in tumor tissues. (C) The quantification of Ki67 score. (D) Western blotting was performed to evaluate the protein levels of phosphorylated-AKT, total-AKT, cleaved-caspase 3, and cleaved-PARP in CRC tumors. The average densitometry readings/intensity ratio was normalized by control and shown below. (F) The quantification of original densitometry readings/intensity ratio. n = 5 for each group. Data represents the analysis and shows mean ± SD. Mel: melatonin treatment; HBO: hyperbaric oxygen; * represents p<0.05; ** indicates p<0.001; *** shows p<0.0001.

To further verify the histological characterization and molecular mechanism in vivo, tumor tissues were analyzed by histological (H&E) and immunohistochemical (Ki67) staining as the Ki67 protein is well recognized as a marker for tumor proliferation. The Ki67 scores obtained demonstrated that 45~70% tumor proliferation was prominently repressed by melatonin, HBO and combined treatments. The result of the present study (Figure 7 A-C). Moreover, AKT activations accompanying with apoptosis induction were significantly inhibited by melatonin, HBO and combined treatments (Figure 7 D-E).

**Figure 8:**
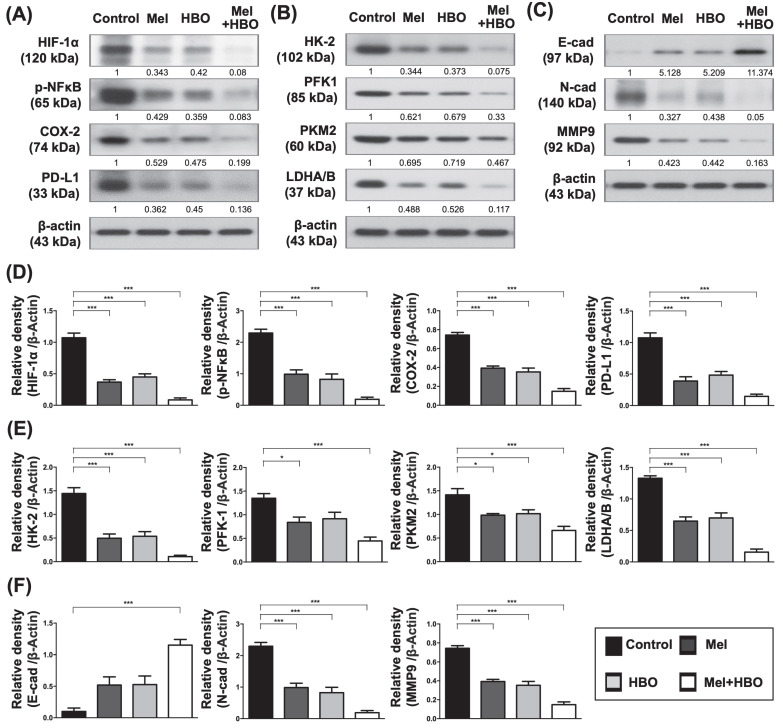
** The effects of melatonin and HBO therapy were down-regulated on multifaceted mechanisms to ameliorate colorectal carcinogenesis.** (A) Western blotting was performed to evaluate the protein levels of HIF-1α, phosphorylated-NFκB, COX-2, and PD-L1 in CRC tumors. (B) Western blotting was performed to evaluate the protein levels of HK-2, phosphofructokinase-1(PFK-1), PKM2, and LDHA/B in CRC tumors. (C) Western blotting was performed to evaluate the protein levels of E-cad (E-cadherin) N-cad (N-cadherin) and MMP9 in CRC tumors. The average densitometry readings/intensity ratio was normalized by control and shown below. (D) The quantification of original densitometry readings/intensity ratio in Fig. 8A. (E) The quantification of original densitometry readings/intensity ratio in Fig. 8B. (F) The quantification of original densitometry readings/intensity ratio in Fig. 8C. n = 5 for each group. Data represents the analysis and shows mean ± SD. Mel: melatonin treatment; HBO: hyperbaric oxygen; * represents p<0.05; ** indicates p<0.001; *** shows p<0.0001.

Furthermore, the tumor inflammatory signaling, HIF-1α, p-NFκB, COX-2, and PD-L1 were significantly down-regulated by melatonin, HBO and combined treatments (Figure 8 A,D). Consistently, the protein levels of HK-2, phosphofructokinase-1(PFK-1), PKM2, and LDHA/B which participated in aerobic glycolysis were significantly retrained by melatonin, HBO and combined treatments (Figure 8B,E). Tumor aerobic glycolysis (i.e., Warburg effect) supports a metabolic environment, which allows for the rapid biosynthesis to provide tumor growth and proliferation 43. In this way, our results revealed these treatments substantially reduced aerobic glycolysis, resulting in blocking the tumor proliferation and tumorigenesis (Figures 6 A-D, 7 A-C, 8 B). On the other hand, the effects of melatonin, HBO and combined treatments significantly up-regulated E-cadherin and significantly down-regulated N-cadherin and MMP9, suggesting these treatments reversed epithelial-to-mesenchymal transition (EMT) process (Figure 8C,F) which is a critical feature of tumor metastasis. Consistent with Figure 6E, up-streams of EMT process, including TGFβ and p-Smad3, were remarkably inhibited by these treatments. Taken together, the effects of melatonin, HBO and combined treatments were clearly delineated to significantly suppress the tumor carcinogenesis through multifaceted mechanisms.

## Discussion

**Figure 9:**
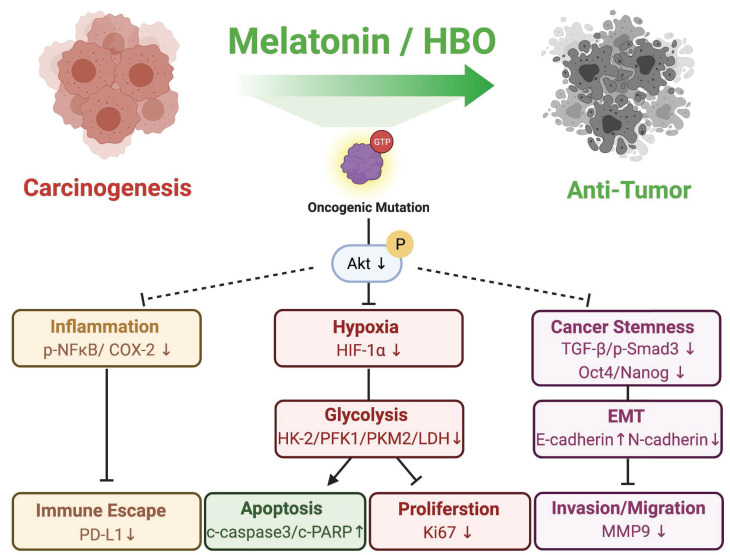
** The schematics illustrating melatonin and HBO treatments effectively inhibited colorectal carcinogenesis through pleiotropic effects and multifaceted mechanisms.** During CRC carcinogenesis, oncogenic mutations induced uncontrolled AKT activation to further stimulate inflammation, hypoxia, glycolysis, cancer stemness generation, and EMT process, followed by malignant phenotypes. Melatonin and HBO treatments significantly suppressed AKT activation to attenuate hypoxia, reduce inflammation, and reverse glycolytic metabolism for restraining immune escape and tumor survival. Additionally, melatonin and HBO treatments repressed cancer stemness via TGF-β/Smad3 signaling axil to inhibit EMT process, migration, and invasion, and finally suppress carcinogenesis to achieve anti-tumor effect. This schematic figure was created with BioRender.com.

To this day, therapeutic efficacies of melatonin and HBO therapies to CRC have been not yet clearly established. To further identify the efficacy of melatonin and HBO therapies in CRC, we evaluated the malignancy phenotypes in vitro and xenografic CRC tumorigenesis in vivo. Our in vivo finding demonstrated that the melatonin and HBO treatments effectively inhibited colorectal carcinogenesis through pleiotropic effects and multifaceted mechanisms, including attenuating hypoxia, reducing inflammation, reversing glycolytic metabolism to restrain tumor survival, repressing migration and invasion via EMT process, ameliorating immune escape, and finally suppressing tumorigenesis (Figure 9), suggesting melatonin and HBO therapy have the potentials to the effective treatments for colorectal cancers.

On the other hand, our in vitro study revealed that melatonin treatment increased apoptosis and decreased many malignancy phenotypes, including cancer growth, colony formation, migration, invasion, and sphere formation. However, HBO therapy did not show these promising capabilities as the melatonin acted on CRC cell lines (Figure 1-5). In vivo study, mice were exposed with HBO therapy twice a week for a total 3 of weeks and the results showed that HBO therapy remarkably suppressed tumorigenesis in vivo, indicating HBO therapy has the therapeutic benefits to CRC. The discrepancy between in vitro and in vivo findings of HBO therapy could be attributed to the following reasons: First, compared to in vivo study, CRC cells were only exposed to HBO therapy once and underwent molecular-cellular levels of studies just after 48 hours of HBO treatment, suggesting that it was only a short-term exposure to HBO. On the other hand, in the in vivo situation, the HBO therapy was followed the practice of clinical setting. Thus, this might adversely lead to a therapeutic effect on inhibiting the CRC cells in vitro (Figure 1-5). Second, cancer cells profoundly interact with the surrounding tumor microenvironment in vivo that would be hard for cell line to entirely mimic tumor microenvironment in vitro. Therefore, HBO therapy was still suggested for its anti-tumor efficacy in CRC.

During CRC carcinogenesis, uncontrollable oncogenic mutations activate a survival signaling pathway. One of the important survival-signaling pathway is PI3K/AKT/mTOR pathway that controls the availability of amino acids and glucose, reduces apoptosis, increases proliferation, stimulates cell growth and finally acquires tumor survival in CRC 51. Additionally, AKT plays as a critical driver of the tumor glycolytic phenotype and triggers ATP generation via multiple mechanisms to gain the bioenergetic capacity and responds to cancer growth. Moreover, the AKT modulates the metabolic alteration by activating transcription factors such as HIF-1, which targets key glycolytic enzyme genes including HK2, PKM2, Pyruvate Dehydrogenase Kinase 1 (PDK1) and LDHA 52, 53. Interestingly, these main glycolytic enzymes not only participate in the Warburg effect but also contribute to tumorigenesis, especially in tumor survival 53. In the present study, CRC cell lines possess oncogenic mutations to activate PI3K/AKT pathway. There has been noted the G13D mutation of KRAS gene and E545K and D549N mutations of PIK3CA gene in DLD-1 cells as well as the G13D and A14V mutations of KRAS gene in LoVo cells 54. Consistent with the above findings 51-54, our study further demonstrated that melatonin and HBO therapies repressed AKT activation induced by oncogenic mutations and then reduced HIF-1 expressions, followed by diminishing the expression of glycolytic enzymes to limit tumor survival and tumorigenesis.

In cancer cells, dysregulation of mitochondrial metabolism always contributes to tumor anabolism, signal transduction, cell survival, and ROS generation 55. Melatonin is synthesized in mitochondria and plays important role on oncostatic effects 56. Study has demonstrated that melatonin treatment effectively inhibits Warburg effect that acts as a reverser to mitochondrial energy metabolism in lung cancer cells 57. In hepatocellular carcinomas patients, melatonin and its metabolic levels are significantly lower than control group and positive correlation of overall survival 58. Most importantly, the percent of disease-control in metastatic CRC patients is significantly elevated in melatonin administration combined with chemotherapy group as compared with merely chemotherapy group 59. These reports indicate that melatonin and its metabolic enzymes may contribute an anti-cancer effect. Indeed, mitochondrial cytochrome P450 1B1 (CP4501B1), which metabolizes melatonin to N-acetylserotonin, can induce tumor apoptosis 56. Consist with previous study 56, 57, our present study further provides the evidence of anti-cancer effect of melatonin, especially in suppression of the glycolysis enzymes levels to reverse Warburg effect.

Metastasis is composed of a multiple-step cell-biological process by which cancer cells disseminate from the primary tumor, then invade the basement membrane, further spread into the circulatory system, and finally settle in secondary tissues/organs such as the liver or lung 60. In aggressive CRC, cancer cells may be through AKT pathway to induce EMT process or generate CSCs accompanying metastasis and even resistant to treatment 61, 62. The accumulating evidence supports that the EMT or CSCs phenotypes play crucial roles for local invasion, distant metastasis, poor prognosis in many types of cancer 63-65. Nevertheless, the malignant tumor microenvironments, such as hypoxia or inflammation, promote the generations of EMT and CSCs through the crossroad of HIF-1 66. Corroborated with the findings of previous studies 63-66, our findings revealed that melatonin and HBO therapies remarkably blocked HIF-1 expression to decrease cancer stemness regulators and reverse EMT process to effectively suppress tumorigenesis in vivo (Figure 6, Figure 8).

It is well known that in the tumor microenvironment, immune surveillance is a crucial control against tumor. Tumor hypoxia and inflammation stimulates cancer progression and cancer immune escape by way of NF‐κB and HIF-1 crosstalk 47, 66. PD-L1 expressed in the tumor surface contributes to an immune checkpoint for preventing immune reaction. Activation of AKT signaling pathways causes PD-L1 induction through HIF-1 transcription factor to facilitate PD-L1 gene transcription 67. On the other hand, Warburg effect results in the accumulation of lactate and protons that leads to acidification in tumor microenvironment, which enhances the malignant phenotypes and causes the immunosuppressive effect 68. According to this theory, it is well discussed and considered whether “Reverse Warburg effect” could improve the tumor microenvironment and restore immune surveillance 69. Accordingly, our findings verified this postulation as previous studies 67-69. Both melatonin and HBO therapies highly restrained the expression of glycolytic enzymes, reversed Warburg effect, attanuated inflamation and hypoxia, as well as improved tumor microenvironment to inhibit the expression of PD-L1 and restrict tumorgenesis (Figure 6-8).

This study has limitations. In fact, in this study, we proposed that combined melatonin and HBO (i.e., cocktail therapy) would be superior to either one alone for reducing colorectal carcinogenesis. Depressingly, according to the evaluation of synergistic effect 70, the combined therapy did not reach a significant synergistic effect than a single therapy on colorectal carcinogenesis. The unexpected discrepancy between our hypothesis and the results could be perhaps due to the following reasons: First, the dosage of melatonin and HBO to be utilized in the present study was based on our previous studies without stepwise titrating their dosages. Accordingly, the optimal dosage of combined therapy that offers the great benefit (i.e., a synergic effect) is currently still unclear. Second, HBO therapy elevates tissue oxygen tension which further triggers the production of reactive oxygen species 71, yet melatonin possesses strong antioxidant effects 11-15. In this way, the cellular effect of HBO therapy may offset by melatonin leading to no synergistic efficacy of this combined therapy. However, our results still provided the anti-tumor evidence of melatonin and HBO therapy through modulating tumor microenvironments to effectively suppress CRC carcinogenesis, which could be considered as a useful therapeutic potential and highlights that it may be applied to cancer treatment in our future clinical practice.

## Conclusion

In conclusion, our findings illustrated new insights into the anti-tumor effect of melatonin and HBO therapies in CRC treatment through the pleiotropic effects and multifaceted mechanisms, including attenuating hypoxia, reducing inflammation, reversing glycolytic metabolism, repressing migration and invasion via EMT process, ameliorating immune escape, and finally suppressing CRC carcinogenesis. This study further highlighted that melatonin and HBO therapies could be novel and potential therapeutic strategies for CRC patients.

## Supplementary Material

Supplementary tables.
