# Supplementary material for: Melatonin and hyperbaric oxygen therapies suppress colorectal carcinogenesis through pleiotropic effects and multifaceted mechanisms
Source: Int J Biol Sci. 2021 Aug 27;17(14):3728–44. doi: 10.7150/ijbs.62280 (PMC8495382; doi:10.7150/ijbs.62280)
Supplement: Supplementary file 1 — Supplementary tables. [file ijbsv17p3728s1.pdf]

Supplementary Materials

Supplementary Table 1. Primer list.
Supplementary Table 2. Western blotting Antibody list.

Supplementary Table 1. Primer list.

Name	Forward primer	Reverse primer
HK-2	TTGACCAGGAGATTGACATGGG	CAACCGCATCAGGACCTCA
PFKM	GGTGCCCGTGTCTTCTTTGT	AAGCATCATCGAAACGCTCTC
PKM2	ATGTCGAAGCCCCATAGTGAA	TGGGTGGTGAATCAATGTCCA
LDAH	ATGGCAACTCTAAAGGATCAGC	CCAACCCCAACAACTGTAATCT
Oct-4	GAGAACCGAGTGAGAGGCAA	CCAGCAGCCTCAAAATCCTC
Slug	AAGCATTTCAACGCCTCCAAA	GGATCTCTGGTTGTGGTATGACA
ABCG2	CAGGTGGAGGCAAATCTTCGT	ACCCTGTTAATCCGTTCGTTTT

Supplementary Table 2. Western blotting Antibody list.

Name	Dilute	Company	Cat No.
cleaved Caspase-3	1:1000	cell signaling	9665S
NQO1	1:1000	Abcam	ab28947
p-AKT	1:1000	cell signaling	9271
Total-AKT	1:1000	cell signaling	9272
PTEN	1:5000	Abcam	ab32199
TGFβ	1:1000	Abcam	ab64715
p-Smad3	1:1000	Abcam	ab28379
Smad3	1:1000	cell signaling	9523
MMP9	1:1000	Abcam	ab76003
HIF-1α	1:1000	Abcam	ab2185
p-NFκB	1:1000	cell signaling	3033
PD-L1	1:2000	Abcam	ab205921
Oct-4	1:1000	Abcam	ab18976
Nanog	1:1000	Abcam	ab106465
cleaved PARP	1:1000	cell signaling	9542S
COX-2	1:1000	Abcam	ab52237
HK-2	1:1000	Abcam	ab209847
PFK1	1:5000	Abcam	ab154804
PKM2	1:1000	cell signaling	4053
LDHA/B	1:1000	cell signaling	3558
E-cadherin	1:1000	Abcam	ab76055
N-cadherin	1:1000	cell signaling	4061S
Actin	1:10000	Merck	MAB1501
